# Synthesis and In Vitro Antiproliferative Activity of New 1-Phenyl-3-(4-(pyridin-3-yl)phenyl)urea Scaffold-Based Compounds

**DOI:** 10.3390/molecules23020297

**Published:** 2018-01-31

**Authors:** Mohammad M. Al-Sanea, Mohammed Safwan Ali Khan, Ahmed Z. Abdelazem, So Ha Lee, Pooi Ling Mok, Mohammed Gamal, Mohamed E. Shaker, Muhammad Afzal, Bahaa G. M. Youssif, Nesreen Nabil Omar

**Affiliations:** 1 Department of Pharmaceutical Chemistry, College of Pharmacy, Aljouf University, Sakaka 2014, Aljouf Province, Saudi Arabia; mgmohamed@ju.edu.sa (M.G.); bgyoussif@ju.edu.sa (B.G.M.Y.); 2 Department of Pharmacology, College of Pharmacy, Aljouf University, Sakaka 2014, Aljouf Province, Saudi Arabia; mshaker2222@yahoo.com (M.E.S.); afzalgufran@gmail.com (M.A.); 3 Department of Pharmacology, Anwarul Uloom College of Pharmacy, Jawaharlal Nehru Technological University, Hyderabad 500001, Telangana, India; 4 Department of Biomedical Sciences, Faculty of Medicine and Health Sciences, Universiti Putra Malaysia, Serdang 43400, Selangor, Malaysia; 5 Department of Biotechnology & Life Sciences, Faculty of Postgraduate Studies for Advanced Sciences, Beni-Suef University, Beni-Suef 62574, Egypt; aznagi2003@gmail.com; 6 Chemical Kinomics Research Center, Korea Institute of Science and Technology, Seoul 136-791, Korea; lsh6211@kist.re.kr; 7 Genetics and Regenerative Medicine Research Centre, Universiti Putra Malaysia, Serdang 43400, Selangor, Malaysia; 8 Department of Pharmaceutical Analytical Chemistry, Faculty of Pharmacy, Beni-Suef University, Alshaheed Shehata Ahmed Hegazy St., Beni-Suef 62574, Egypt; 9 Department of Pharmacology and Toxicology, Faculty of Pharmacy, Mansoura University, Mansoura 35516, Egypt; 10 Department of Pharmaceutical Organic Chemistry, Faculty of Pharmacy, Assiut University, Assiut 71526, Egypt; 11 Department of Biochemistry, Faculty of Pharmacy, Modern University for Technology & Information, 11571 Cairo, Egypt; dr.n.nabil@gmail.com

**Keywords:** cancer, cell line, synthesis, urea derivatives, antiproliferative, activity

## Abstract

A new series of 1-phenyl-3-(4-(pyridin-3-yl)phenyl)urea derivatives were synthesized and subjected to in vitro antiproliferative screening against National Cancer Institute (NCI)-60 human cancer cell lines of nine different cancer types. Fourteen compounds **5a**–**n** were synthesized with three different solvent exposure moieties (4-hydroxylmethylpiperidinyl and trimethoxyphenyloxy and 4-hydroxyethylpiperazine) attached to the core structure. Substituents with different π and σ values were added on the terminal phenyl group. Compounds **5a**–**e** with a 4-hydroxymethylpiperidine moiety showed broad-spectrum antiproliferative activity with higher mean percentage inhibition values over the 60-cell line panel at 10 µM concentration. Compound **5a** elicited lethal rather than inhibition effects on SK-MEL-5 melanoma cell line, 786-0, A498, RXF 393 renal cancer cell lines, and MDA-MB-468 breast cancer cell line. Two compounds, **5a** and **5d** showed promising mean growth inhibitions and thus were further tested at five-dose mode to determine median inhibitory concentration (IC_50_) values. The data revealed that urea compounds **5a** and **5d** are the most active derivatives, with significant efficacies and superior potencies than paclitaxel in 21 different cancer cell lines belonging particularly to renal cancer and melanoma cell lines. Moreover, **5a** and **5d** had superior potencies than gefitinib in 38 and 34 cancer cell lines, respectively, particularly colon cancer, breast cancer and melanoma cell lines.

## 1. Introduction

Cancer in its essence is a genetic disease; accumulation of inherited and/or acquired defects in cell proliferation and survival regulatory genes is responsible for cancer precipitation [1]. Oncogenes, tumor-suppressor genes and stability genes are the three types of genes in which variations are the possible causes of cancer. These defects are required for a clinically significant cancer to occur and drive transformation of normal cell into cancerous ones [2]. Despite the availability of developed drugs including targeted tumor therapies, the World Health Organization (WHO) has announced the great possibility that the universal cancer burden will increase by 15 million new cases per year by 2020, unless further preventive measures are considered. The developments of new anticancer drugs signify a major objective and challenge for modern medicinal chemistry.

The urea chemotype is one of the most interesting scaffold-based compounds in the treatment of cancer diseases [3]. Apart from anticancer activity, other biological activities have been reported for urea derivatives such as antidiabetic [4,5,6], antitubercular [7,8,9], antimicrobial [10,11,12], and anti-inflammatory activities [10,11,13]. Much attention has been paid to the chemistry and biological activities of the diarylurea nucleus. Several compounds possessing diarylurea scaffolds have been recently reported as potential antiproliferative agents [12,14,15,16,17,18,19,20].

In the present study, a new series of diarylurea derivatives possessing 1-phenyl-3-(4-(pyridin-3-yl)phenyl)urea moieties were designed, synthesized and tested for their in vitro antiproliferative activities against National Cancer Institute (NCI)-60 cancer cell lines. Various substituted terminal phenyl moieties were introduced to investigate the influence of electronic and hydrophobic effects on the antiproliferative activity of the titled compounds. Furthermore, three hydrogen bondable moieties were introduced to the internal phenyl moiety to explore whether the addition of such moieties would result in a significant increase in anticancer activity.

## 2. Results and Discussion

### 2.1. Chemistry

**Scheme 1:**
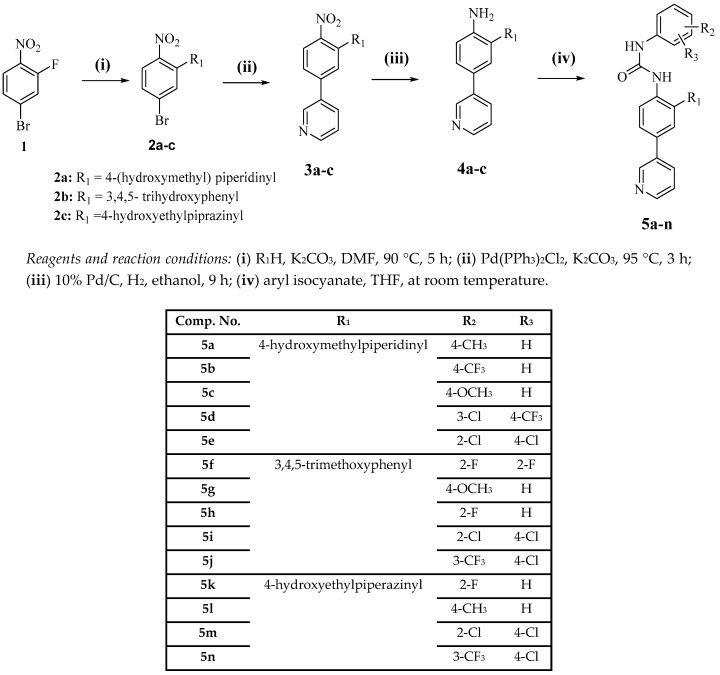
Synthetic scheme of diarylurea derivatives **5a**–**n.**

Synthesis of the target compounds **5a**–**n** was achieved through the pathway illustrated in Scheme 1. Regioselective nucleophilic aromatic substitution of 2-fluoro-4-bromonitrobenzene (**1**) with three hydrogen bondable moieties (4-hydroxylmethylpiperidinyl or trimethoxyphenyloxy or 4-hydroxyethylpiperazine) was the first step. The nitro group in 2-fluoro-4-bromonitrobenzene increases the reactivity of the aryl halide by decreasing the energy of the transition state according to the Hammond postulates and stabilizes the intermediate carbanion, furthermore the fluorine atom is more electronegative and then its reactivity is much higher than that of the bromine atom. Therefore, the substitution reaction was directed to the *ortho* position of the nitro group rather than the *para* position forming monosubstituted nitrobenzenes **2a**–**c** (Scheme 1).

The negatively polarized carbon-metal bond is well suited for the purpose of carbon-carbon forming reactions. Monosubstituted nitrobenzenes **2a**–**c** were subjected to coupling reactions with 3-pyridineboronic acid in the presence of bis(triphenylphosphine)palladium(II) dichloride to afford disubstituted nitrobenzenes **3a**–**c**.

The structure of the newly synthesized compounds **3a**–**c** was confirmed on the basis of ^1^H-NMR and ^13^C-NMR spectroscopic data. The ^1^H-NMR spectra of compound **3c** exhibited triplet signals at *δ* 2.67 ppm and 3.69 ppm (2H) corresponding to NCH_2_CH_2_OH. Triplet signals at *δ* 2.748 ppm and 3.21 ppm (4H) correspond to the piperazine moiety (NCH_2_CH_2_N)_2_ grouping. In addition, characteristic signals corresponding to aromatic protons were observed. The newly formed disubstituted nitrobenzenes **3a**–**c** were subjected to palladium-catalyzed reduction to the corresponding amines **4a**–**c**. The ^1^H-NMR spectra of compounds **4a**–**c** exhibited a multiplet signal at *δ* 7.29–7.30 ppm (2H) corresponding to the NH_2_ group. Finally, nucleophilic attach of the anilinic amino group on the electrophilic carbon of a phenyl isocyanate group was followed to successfully get the final target diaryl urea compounds **5a**–**n** in yields between 64% and 91%. The ^1^H-NMR of all final compounds **5a**–**n** showed additional signals at *δ* value ranges of 7–9 ppm assigned to the terminal phenyl moiety added through the final last step of the synthetic scheme through reaction of different arylisocyanates with the semifinal compounds **4a**–**c**. Moreover, the ^1^H-NMR spectra of the final urea derivatives **5a**–**n** indicated the presence of two broad singlets appeared at around *δ* 9.54 ppm for NH proton attached to the internal phenyl ring whereas other NH proton attached to terminal ring appeared at around *δ* 8.72 ppm.

### 2.2. In Vitro Antiproliferative Activities against the NCI-60 Cell Line Panel

#### 2.2.1. Single Dose Testing

The newly synthesized target compounds **5a**–**n** were submitted to National Cancer Institute (NCI, Bethesda, ML, USA; www.dtp.nci.nih.gov., accessed on 4 December 2017), and the seven compounds **5a**–**g** shown in Table 1 were selected on the basis of their degree of structural variation and computer modeling techniques for evaluation of their antineoplastic activity. The selected compounds were subjected to in vitro anticancer assay against tumor cells in a full panel of 60 cell lines taken from nine different tissues (blood, lung, colon, CNS, skin, ovary, kidney, prostate, and breast). The compounds were tested at a single-dose concentration of 10 µM, and the percentages of growth inhibition over the 58 tested cell lines were determined.

Upon comparing the effect of hydrogen bondable moieties directly attached to internal phenyl ring on activity, it was found that compounds **5a**–**e** possessing a 4-hydroxymethylpiperidine group were more active than compounds **5f**–**g** with a 3,4,5-trimethoxyphenol. This directly reflects the influences of such moieties on the antiproliferative activity of the title scaffold. The mean % growth of the NCI-60 cancer cell line panel after treatment with each of the tested compounds at 10 µM is listed in Table 1.

Compounds **5a**, **5d** and **5e** showed higher antiproliferative activity than the corresponding *p*-methoxyphenyl and *p*-trifluoromethylphenyl analogues **5b** and **5c**. It was clear that compounds with trimethoxyphenyl derivatives are not suitable as potential anticancer agents, while those having a piperidin-4-yl methanol moiety are promising anticancer agents. The average growth percentage of compounds **5a**, **5d** and **5e** which have a piperidin-4-yl methanol are 22.16%, 24.67% and 32.41%, respectively (Table 2). Compounds **5d** and **5e** with disubstituted terminal phenyl rings have superior activity than the corresponding monosubstituted derivatives against most of leukemia cell lines. Compound **5a** with an electron-donating group (methyl) showed lethal effects on the SK-MEL-5 melanoma cell line, MDA-MB-468 breast cancer cell line and three renal cancer cell lines (786-0, A498 and RXF 393). Therefore, it can be concluded that the antiproliferative activity of the tested compounds against diverse cancer cell lines differs with the different steric and/or electronic properties of the substituents on the terminal phenyl moiety.

**Figure 1:**
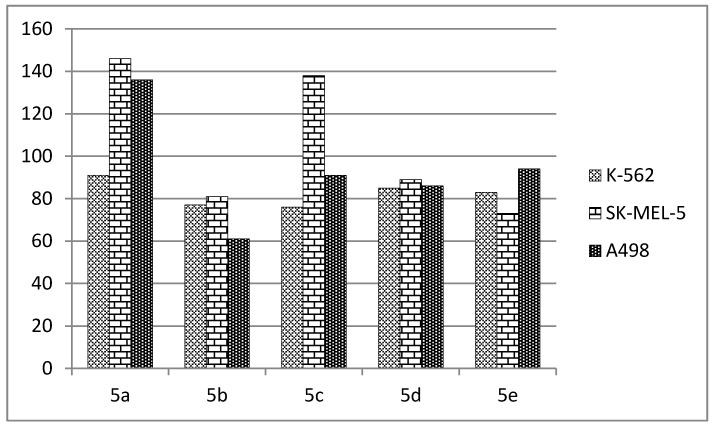
The percentage inhibition values of compounds **5a**–**d** against SK-MEL-5, A498 and K-562 cell lines.

The percentage inhibition values of the most active compounds against the most sensitive cell lines are summarized in Table 2. SK-MEL-5 melanoma cell line was the most sensitive cell line to this series of compounds. The most active compounds against SK-MEL-5 were **5a** and **5c** with percentage inhibitions of 146.1% and 138.3%, respectively. K-562 leukemia and A498 renal cancer cell lines were also sensitive to the compounds. Compounds **5a**–**e** were active against three cell lines (K-562, SK-MEL-5, A498), while compounds **5a** and **5c** showed higher potency over SK-MEL-5 (Figure 1). Among the seven target compounds, compound **5a** and **5d** showed the most promising results. They exerted broad-spectrum antiproliferative activity against different cell lines of different cancer types, including leukemia, colon, renal and breast cancers. Therefore, these compounds could be potential leads for the future development of broad-spectrum anticancer agents.

**Figure 2:**
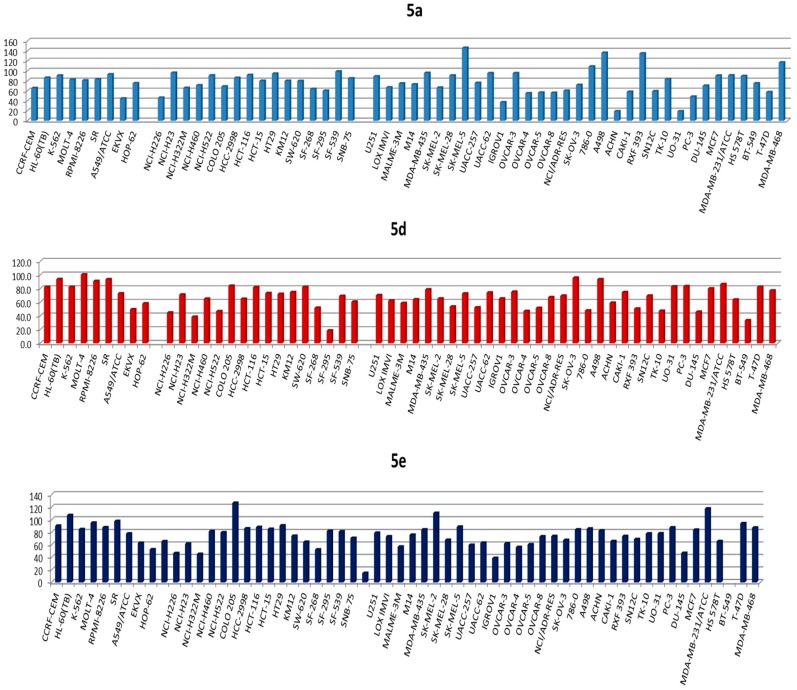
Percentage growth of 60 cell line panel upon treatment with compounds **5a**, **5d** and **5e** at 10 µM.

Among all the tested derivatives, compounds **5a**, **5d**, and **5e** showed the highest mean inhibitions. The percentages of inhibition of these three compounds over each tested cell line of the NCI-60 panel at 10-µM concentration are depicted in Figure 2.

**Figure 3:**
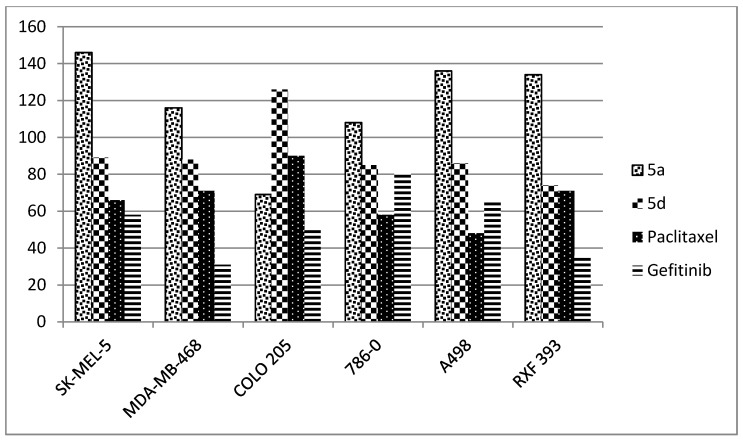
Comparison of percentage inhibition values expressed by **5a**, **5d**, paclitaxel and gefitinib against the most sensitive cell lines.

The three compounds **5a**, **5d** and **5e** exerted broad-spectrum antiproliferative activities against the NCI-60 human cancer cell lines of different cancer types. Among them, compound **5a** was the most active as it showed the highest percentage inhibition values with more than 70% inhibition against SR leukemia cell line, SK-MEL-5 and UACC-257 melanoma cell lines, and T-47D and MDA-MB-468 breast cancer cell lines. The results of compound **5a** and **5d** against five cell lines (SK-MEL-5, MDA-MB-468, 786-0, COLO 205 and RXF 393) were compared with paclitaxel and gefitinib as reference standard drugs, as illustrated in Figure 3. The results of paclitaxel and gefitinib were obtained from the NCI data warehouse index.

#### 2.2.2. Five Dose Testing

Compounds **5a** and **5d** with promising results in single-dose test and satisfying the criteria set by the NCI for activity in that preliminary assay were further tested in a five dose testing mode at 10-fold dilution (100–0.01 µM) on the full panel. For each of these compounds, three response parameters; the IC_50_, the concentration producing 50% growth inhibition (GI), a measure of compound potency), TGI (the concentration producing 100% GI, a measure of compound efficacy) and LC_50_ (the concentration causing 50% lethality, a measure of compound efficacy and cytotoxicity) were determined. The two tested compounds **5a** and **5d** showed high potency with one-digit micro molar IC_50_ values over most of the cell lines.

Compounds **5a** and **5d** satisfied the pre-determined threshold growth inhibition criteria and were further selected for NCI full panel five dose assay at 10-fold dilutions of five different concentrations (0.01, 0.1, 1, 10 and 100 µM).

**Figure 4:**
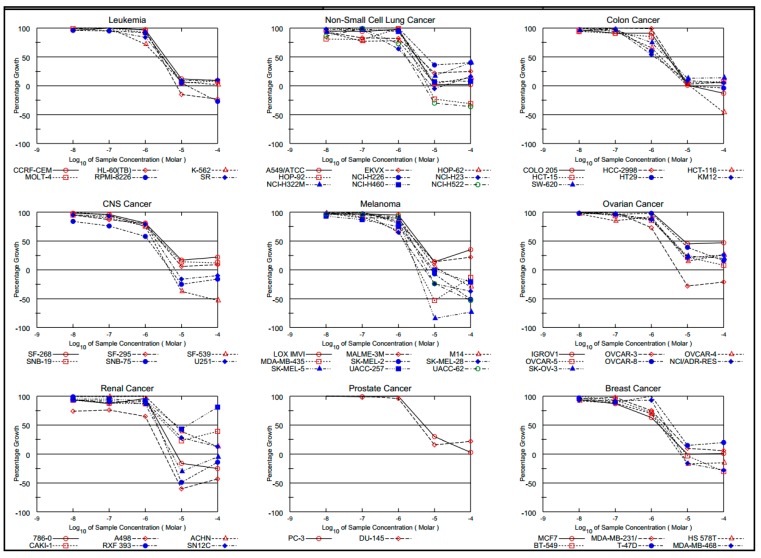
Dose–antiproliferative response of compound **5a** against nine different cancer cell lines**.**

The result of tested compound **5a** is given by three response parameters (GI_50_, TGI and LC_50_) for each cell line from log concentration vs. percentage growth inhibition curves on cell lines derived from nine cancers (Figure 4).

Compounds **5a** and **5d** showed remarkable broad-spectrum potency against multiple other cell lines in the range of IC_50_ values of 1.25–8.44 and 1.26–3.75 µM, respectively. Among the 60 cancer cell lines, **5a** compound exhibited significant inhibition against colon KM12 (IC_50_: 1.25 µM), CNS SNB-75 (IC_50_: 1.26 µM), melanoma MDA-MB-435 (IC_50_: 1.41 µM), melanoma SK-MEL-28 (IC_50_: 1.49 µM) and renal A498 (IC_50_:1.33 µM). The five cell lines against which compound **5d** was found to be effective are non-small cell lung cancer EKVX (IC_50_: 1.72 µM), non-small cell lung cancer H522 (IC_50_: 1.73 µM), colon COLO 205 (IC_50_: 1.65 µM), melanoma SK-MEL-5 (IC_50_: 1.63 µM) and renal A498 (IC_50_: 1.26 µM). By referring to the efficacy parameter (TGI values) of the target compounds **5a** and **5d**, it was demonstrated that the most potent compound **5a** was efficacious towards A498 renal, RXF 393 renal cancer cells, SF-539 CNS cancer cell, an d NCI-H522 non- small cell lung cancer cell line with TGI values of 3.32, 4.69, 4.67, and 5.12 µM, respectively, while compound **5d** exerted remarkable efficacies against NCI-H522, COLO 205, LOX IMVI, SK-MEL-5 and RXF 393 cell lines, being able to induce TGI at concentrations below 3.66 µM and 50% lethality (LC_50_) at concentrations below 6.96 µM.

## 3. Materials and Methods

### 3.1. General Information

The NMR spectra were recorded with a Bruker spectrometer (Billerica, MA, USA), operating at 400 MHz for ^1^H-NMR and 100 MHz for ^13^C-NMR. The multiplicities were abbreviated as s: singlet, d: doublet, t: triplet, m: multiplet, q: quartet. The coupling constants *J* are recorded in Hertz (Hz) and are liable to a little difference because they used the integral values measured by the spectrometer. The relative shift values of peak are recorded by ppm unit using tetramethylsilane (TMS) as standard material. Melting points were determined on a SRS OPTIMELT apparatus (Stanford Research Systems, Sunnyvale, CA, USA). The FT-IR spectra were obtained on a 16E PC FT-IR spectrometer (Perkin Elmer, Waltham, MA, USA). Thin layer chromatography (TLC) was performed using precoated plates (0.25 mm, Merck, Billerica, MA, USA) of silica gel 60 F_254_ (230~400 mesh) for monitoring all reactions and under ultraviolet irradiation (254 nm). Column chromatography separations are performed using silica gel (230~400 mesh, Merck). All the commercially available reagent chemicals were obtained from Sigma-Aldrich Corporation (St. Louis, MO, USA), Tokyo Chemical Industry Company Limited (Fukaya, Tokyo, Japan), Wako Pure Chemical Industries (Chuo-Ku, Osaka, Japan), Acros (Jersey City, NJ, USA) and Dae-Jung Chemicals (Shiheung, Gyeonggi, Korea), and generally used without further purification.

### 3.2. General Method for Synthesis of Compounds ***2a**–**c***

4-Bromo-2-fluoro-1-nitrobenzene (0.50 g, 2.28 mmol), piperidin-4-ylmethanol, 2-(piperazin-1-yl)ethanol and 3,4,5-trimethoxyphenol (2.28 mmol), K_2_CO_3_ (0.32 g, 2.28 mol) were mixed in DMF (10 mL) and heated at 90 °C under N_2_ for 5 h. After cooling, the reaction mixture was filtered to remove solid and the solvent was evaporated. The residue was dissolved in dichloromethane and washed with water, dried over anhydrous MgSO_4_ and concentrated *in vacuo*. The residue solid was applied on column of silica gel and then eluted with the mixed solvent of ethyl acetate and hexanes (3:1, *v*/*v*) to give the pure product as a white solid.

*1-(5-Bromo-2-nitrophenyl)piperidin-4-yl)methanol* (**2a**). Yield: 0.61 g (85%), ^1^H-NMR (CDCl_3_) *δ* 1.50–1.53 (m, 2H), 1.72 (s, 1H), 1.87 (d, *J* = 12.8 Hz, 2H), 2.89 (t, *J* = 12.4 Hz, 2H), 3.34 (d, *J* = 12.4 Hz, 2H), 3.60 (d, *J* = 6.4 Hz, 2H), 7.10 (d, *J* = 8.8 Hz, 1H), 7.27 (s, 1H), 7.69 (d, *J* = 8.8 Hz, 1H).

*2-(4-(5-Bromo-2-nitrophenyl)piperazin-1-yl)ethan-1-ol* (**2b**)*.* Yield: 0.7 g (93%). ^1^H-NMR (CDCl_3_) *δ* 2.661 (t, *J* = 5.2 Hz, 2H), 2.714 (t, *J* = 4.8 Hz, 4H), 3.136 (t, *J* = 4.8 Hz, 4H), 3.688 (t, *J* = 5.2 Hz, 2H), 7.164 (d, *J* = 8.8 Hz, 1H), 7.274 (s, 1H), 7.70 (d, *J* = 8.8 Hz, 1H).

*5-(5-Bromo-2-nitrophenoxy)-1,2,3-trimethoxybenzene* (**2c**). Yield: 0.8 g (92%). ^1^H-NMR (CDCl_3_) *δ* 3.72 (s, 6H), 3.78 (s, 3H), 6.33 (s, 2H), 7.09 (d, *J* = 8.8 Hz, 1H), 7.31 (s, 1H), 7.68 (d, *J* = 8.8 Hz, 1H).

### 3.3. General Method for Synthesis of Compounds ***3a**–**c***

Compounds **2a**–**c**, 3-pyridylboronic acid (120 mole %) Pd_2_(PPh_3_)_2_Cl_2_ (5 mole %) and K_2_CO_3_ (200 mole %) were mixed and dissolved in degassed mixed solvent of acetonitrile and water (4:1, *v*/*v*). The mixture was bubbled with nitrogen for 15 min and then heated at 95 °C for 3 h. After being cooled to room temperature, the mixture was diluted with water and extracted with ethyl acetate (3 × 5 mL). The organic layers were combined, dried over anhydrous MgSO_4_, and concentrated *in vacuo*. The residue was then subjected to flash chromatography using the appropriate ratios of hexanes and ethyl acetate as mobile phases.

*1-(2-Nitro-5-(pyridin-3-yl)phenyl)piperidin-4-yl)methanol* (**3a**). Yield: 0.42 g (87%). ^1^H-NMR (CDCl_3_) *δ* 1.50–1.53 (m, 2H), 1.72 (s, 1H), 1.87 (d, *J* = 12.8 Hz, 2H), 2.95 (t, *J* = 12 Hz, 2H), 3.41 (d, *J* = 12.4 Hz, 2H), 3.61 (d, *J* = 6.4 Hz, 2H), 7.18 (dd, *J* = 1.6 and 8.4 Hz, 1H), 7.29 (s, 1H), 7.49 (dd, *J* = 4.8 and 8 Hz, 1H), 7.96 (s, 1H), 7.93 (s, 1H), 8.70 (d, *J* = 4.8 Hz, 1H), 8.87 (s, 1H).

*3-(4-Nitro-3-(3,4,5-trimethoxyphenoxy)phenyl)pyridine* (**3b**). Yield: 0.66 g (83%). ^1^H-NMR (CDCl_3_) *δ* 3.75 (s, 6H), 3.75 (s, 3H), 6.34 (s, 2H), 7.14 (d, *J* = 2.0 Hz, 1H), 7.32–7.35 (m, 2H), 7.75 (td, *J* = 8.0 and 2.0 Hz, 1H), 7.98 (d, *J* = 8.0 Hz, 1H), 8.55 (dd, *J* = 5.2 and 2.0 Hz, 1H), 8.68 (d, *J* = 2.0 Hz, 1H).

*2-(4-(2-Nitro-5-(pyridin-3-yl)phenyl)piperazin-1-yl)ethan-1-ol* (**3c**). Yield: 0.60 g (86%). ^1^H-NMR (CDCl_3_) *δ* 2.67 (t, *J* = 5.2 Hz, 2H), 2.748 (t, *J* = 4.8 Hz, 4H), 3.21 (t, *J* = 4.8 Hz, 4H), 3.69 (t, *J* = 5.2 Hz, 2H), 7.24 (dd, *J* = 8.4 and 1.6 Hz, 1H), 7.29 (s, 1H), 7.45 (dd, *J* = 4.8 and 8 Hz, 1H), 7.90 (dt, *J* = 1.6 and 8 Hz, 1H), 7.94 (d, *J* = 8.4 Hz, 1H), 8.70 (dd, *J* = 1.6 and 4.8 Hz, 1H), 8.86 (d, *J* = 1.6 Hz, 1H).

### 3.4. General Method for the Palladium Catalyzed Reduction of Nitrobenzenes ***3a**–**c*** to the Corresponding Anilines ***4a**–**c***

To a solution of compound **3a**–**c** (0.5 g, 22.6 mmol) in ethanol (150 mL), (0.05 g) of 10% Pd/C was added. The reaction mixture was stirred at room temperature under an atmosphere of hydrogen for 9 h. After completion of the reaction, the resulting mixture was filtered through celite, and the filtered catalyst was washed with ethanol. The filtrate was concentrated under vacuum to afford compound **4a**–**c** which was used in the next step without further purification.

*(1-(2-Amino-5-(pyridin-3-yl)phenyl)piperidin-4-yl)methanol* (**4a**). Yield: 0.39 g (88%). ^1^H-NMR (CDCl_3_) *δ* 1.50–1.54 (m, 2H), 1.69 (s, 1H), 1.92 (d, *J* = 12.4 Hz, 2H), 2.72 (t, *J* = 9.6 Hz, 2H), 3.26 (d, *J* = 11.2 Hz, 2H), 3.63 (t, *J* = 2.8 Hz, 2H), 6.85(d, *J* = 8 Hz, 1H), 7.29–7.31 (m, 3H), 7.85 (d, *J* = 8 Hz, 1H), 8.51 (d, *J* = 4.8 Hz, 1H), 8.82 (d, *J* = 2.8 Hz, 1H).

*4-(Pyridin-3-yl)-2-(3,4,5-trimethoxyphenoxy)aniline* (**4b**). Yield: 0.42 g (91%). ^1^H-NMR (CDCl_3_) *δ* 3.74 (s, 6H), 3.77 (s, 3H), 6.34 (s, 2H), 6.88(d, *J* = 8.0 Hz, 1H), 7.30–7.33 (m, 3H), 7.86 (d, *J* = 8 Hz, 1H), 8.49 (d, *J* = 4.8 Hz, 1H), 8.79 (d, *J* = 2.8 Hz, 1H).

*2-(4-(2-Amino-5-(pyridin-3-yl)phenyl)piperazin-1-yl)ethanol* (**4c**). Yield: 0.40 g (88%). ^1^H-NMR (CDCl_3_) *δ* 2.67 (t, *J* = 5.2 Hz, 2H), 2.748 (t, *J* = 4.8 Hz, 4H), 3.21 (t, *J* = 4.8 Hz, 4H), 3.69 (t, *J* = 5.2 Hz, 2H), 6.79 (d, *J* = 8 Hz, 1H), 7.15 (dd, *J* = 8.0 and 1.6 Hz, 1H), 7.20 (s, 1H), 7.29 (dd, *J* = 4.8 and 1.6 Hz, 1H), 7.77 (dt, *J* = 1.6 and 8.0 Hz, 1H), 8.46 (dd, *J* = 1.6 and 4.8 Hz, 1H), 8.76 (d, *J* = 1.6 Hz, 1H).

### 3.5. General Procedure for the Synthesis of Urea Derivatives ***5a**–**n***

To a solution of aryl isocyanate (0.2 mmol) in THF (1 mL), the appropriate aniline compounds **4a**–**c** (0.2 mmol) were added. The mixture was stirred at room temperature until arylisocyanate was completely reacted. The solvent was removed *in vacuo*, and the residue was then subjected to flash chromatography using the appropriate ratios of hexanes and ethyl acetate as mobile phases to obtain urea compounds (**5a**–**n**).

*1-(2-(4-(Hydroxymethyl)piperidin-1-yl)-4-(pyridin-3-yl)phenyl)-3-(4-methylphenyl)urea* (**5a**). Yield: 0.045 g (64%). m.p.: 215–217 °C. IR (KBr) 3663, 3315, 3185, 1678, 1519, 838 cm^−1^. ^1^H-NMR (DMSO*-d*_6_) *δ* 1.53 (s, 2H), 1.80 (s, 2H), 2.27 (s, 3H), 2.74 (t, *J* = 10.0 Hz, 2H), 3.03 (s, 2H), 3.4 (s, 2H), 4.56 (t, *J* = 5.2 Hz, 1H), 7.11 (s, 1H), 7.13 (s, 1H), 7.39 (s, 1H), 7.41 (s, 1H), 7.45–7.48 (m, 2H), 7.49 (d, *J* = 2 Hz, 1H), 8.05 (td, *J* = 2.0 and 8.4 Hz, 1H), 8.11 (s, 1H), 8.18 (d, *J* = 8.4 Hz, 1H), 8.52 (dd, *J* = 1.2 and 4.4 Hz, 1H), 8.89 (d, *J* = 2.0 Hz, 1H), 9.54 (s, 1H).^13^C-NMR (DMSO-*d*_6_) *δ* 20.83, 29.46, 38.66, 52.62, 66.48, 119.07, 119.17, 119.88, 122.92, 124.20, 129.7, 130.98, 131.31, 134.03, 134.65, 136.00, 137.64, 143.39, 147.79, 148.26, 152.97.

*1-(2-(4-(Hydroxymethyl)piperidin-1-yl)-4-(pyridin-3-yl)phenyl)-3-(4-(trifluoromethyl) phenyl)thiourea* (**5b**). Yield: 0.07 g (86%). m.p.: 98–100 °C. IR (KBr) 3580, 3210, 3041, 2926, 1590, 1520, 838 cm^−1^. ^1^H-NMR (CD_3_OD) *δ* 1.23–1.25 (m, 2H), 1.59 (s, 1H), 1.81 (d, *J* = 10.8, 2H), 2.76 (t, *J* = 10 Hz, 2H), 3.13 (d, *J* = 12.0 Hz, 2H), 3.37 (d, *J* = 6.4 Hz, 2H), 7.39 (dd, *J* = 2.0 and 8.4 Hz, 1H), 7.44 (d, *J* = 2.0 Hz, 1H), 7.52 (dd, *J* = 5.2 and 8.0 Hz, 1H), 7.70 (d, *J* = 8.4 Hz, 2H), 7.76 (d, *J* = 8.4 Hz, 2H), 8.09 (dt, *J* = 1.6 and 8.0 Hz, 1H), 8.33 (d, *J* = 8.4 Hz, 1H), 8.51 (dd, *J* = 1.2 and 4.8 Hz, 1H), 8.80 (d, *J* = 2 Hz, 1H). ^13^C-NMR (CD_3_OD) *δ* 29.18, 38.04, 51.94, 66.44, 118.47, 121.85, 123.75, 124.10, 124.24, 125.82, 126.74, 133.22, 134.31, 135.04, 136.80, 142.29, 146.04, 146.67, 147.09, 178.85.

*1-(2-(4-(Hydroxymethyl)piperidin-1-yl)-4-(pyridin-3-yl)phenyl)-3-(4-methoxyphenyl)urea* (**5c**). Yield: 0.061 g (84%). m.p.: 205–207 °C. IR (KBr) 3585, 3314, 3196, 2927, 1673, 1506, 835 cm^−1^. ^1^H-NMR (DMSO*-d*_6_) *δ* 1.51–1.54 (m, 3H), 1.80 (d, *J* = 9.6 Hz, 2H), 2.74 (t, *J* = 10.0 Hz, 2H), 3.02 (d, *J* = 11.2 Hz, 2H), 3.37 (s, 2H), 3.74 (s, 3H), 4.55 (t, *J* = 5.2 Hz, 1H), 6.90 (s, 1H), 6.92 (s, 1H), 7.41–7.42 (m, 1H), 7.43–7.46 (m, 1H), 7.46–7.48 (m, 2H), 7.49 (d, *J* = 2.0 Hz, 1H), 8.04 (t, *J* = 2.4 Hz, 1H), 8.06 (s, 1H), 8.19 (d, *J* = 8.8 Hz, 1H), 8.52 (dd, *J* = 1.2 and 4.8 Hz, 1H), 8.89 (d, *J* = 2.0 Hz, 1H), 9.41 (s, 1H). ^13^C-NMR (DMSO*-d*_6_) *δ* 29.50, 38.64, 52.67, 55.64, 66.48, 114.56, 119.10, 119.68, 121.10, 122.98, 124.20, 130.85, 133.11, 134.01, 134.78, 136.00, 143.25, 147.78, 148.24, 153.12, 155.18.

*1-(2-(4-(Hydroxymethyl)piperidin-1-yl)-4-(pyridin-3-yl)phenyl)-3-(3-chloro-4-trifluoromethyl-phenyl)urea* (**5d**). Yield: 0.072 g (85%). m.p.: 170–173 °C. IR (KBr) 3657, 3298, 3120, 2935, 1679, 1548, 804 cm^−1^. ^1^H-NMR (DMSO*-d*_6_) *δ* 1.55 (s, 2H), 1.81 (s, 2H), 2.74 (s, 2H), 3.03 (s, 2H), 3.4 (s, 2H), 4.56 (t, *J* = 5.2 Hz, 1H), 7.44 (d, *J* = 2.0, 1H), 7.46 (s, 1H), 7.53 (d, *J* = 1.6 Hz, 1H), 7.64 (s, 1H), 7.72 (s, 1H), 8.06 (s, 1H), 8.12 (d, *J* = 2.0 Hz, 1H), 8.17 (d, *J* = 8.4 Hz, 1H), 8.21 (s, 1H), 8.54 (dd, *J* = 1.2 and 4.4 Hz, 1H), 8.89 (d, *J* = 2.0 Hz, 1H). ^13^C-NMR (DMSO*-d*_6_) *δ* 29.51, 38.64, 52.67, 66.45, 117.13, 119.23, 120.06, 123.01, 123.33, 124.20, 131.65, 132.56, 133.98, 134.11, 135.89, 139.90, 143.64, 147.84, 148.38, 152.67.

*1-(2-(4-(Hydroxymethyl)piperidin-1-yl)-4-(pyridin-3-yl)phenyl)-3-(2,4-dichlorophenyl)urea* (**5e**). Yield: 0.062 g (78%). m.p.: 189–191 °C. IR (KBr) 3582, 3305, 3097, 2924, 1677, 1587, 874 cm^−1^. ^1^H-NMR (DMSO*-d*_6_) *δ* 1.55 (s, 3H), 1.81 (s, 2H), 2.74 (s, 2H), 3.04 (s, 2H), 3.4 (s, 2H), 4.56 (t, *J* = 5.2 Hz, 1H), 7.37 (dd, *J* = 2.0 and 8.8 Hz, 1H), 7.44–7.46 (m, 2H), 7.54–7.56 (m, 2H), 7.95 (d, *J* = 2.4 Hz, 1H), 8.05 (d, 1H), 8.18–8.19 (m, 2H), 8.53 (d, *J* = 4 Hz, 1H), 8.89 (s, 1H), 9.96 (s, 1H). ^13^C-NMR (DMSO*-d*_6_) *δ* 29.49, 38.64, 52.65, 66.46, 118.74, 119.17, 119.79, 120.10, 122.97, 123.62, 124.19, 131.08, 131.58, 134.03, 134.09, 135.91, 140.51, 143.64, 147.83, 148.35, 152.61.

*1-(2,4-Difluorophenyl)-3-(4-(pyridin-3-yl)-2-(3,4,5-trimethoxyphenoxy)phenyl)urea* (**5f**). Yield: 0.201 g (70%). m.p.: 165–167 °C. IR (KBr) 3291, 3039, 2937, 1710, 1597, 844 cm^−1^. ^1^H-NMR (DMSO*-d*_6_) *δ* 3.67 (s, 3H), 3.752 (s, 6H), 6.50 (s, 2H), 7.076 (t, *J* = 8.4 Hz, 1H), 7.19 (d, *J* = 2.0 Hz, 1H), 7.32 (dt, *J* = 2.8 and 11.6 Hz, 1H), 7.43 (dd, *J* = 8.0 and 4.8 Hz, 1H), 7.43 (dd, *J* = 2.4 and 8.8 Hz, 1H), 7.96 (td, *J* = 8.8 and 1.6 Hz, 1H), 8.19–8.21 (m, 1H), 8.40 (d, *J* = 8.8 Hz, 1H), 8.51 (dd, *J* = 4.8 and 1.6 Hz, 1H), 8.79 (d, *J* = 2.4 Hz, 1H). ^13^C-NMR (DMSO*-d*_6_) *δ* 56.55, 60.62, 97.48, 103.98, 104.24, 104.49, 111.39, 111.58, 115.93, 120.70, 122.51, 124.28, 129.25, 131.02, 131.63, 131.98, 134.12, 134.57, 135.23, 146.61, 147.72, 148.61, 152.67, 154.16.

*1-(4-Methoxyphenyl)-3-(4-(pyridin-3-yl)-2-(3,4,5-trimethoxyphenoxy)phenyl)urea* (**5g**). Yield: 0.11 g (77.5%). m.p.: 180–183 °C. IR (KBr) 3330, 3059, 2938, 1705, 1597, 830 cm^−1^. ^1^H-NMR (DMSO*-d*_6_) *δ* 3.67 (s, 3H), 3.73 (s, 3H), 3.75 (s, 6H), 6.49 (s, 2H), 6.89 (s, 1H), 6.91 (s, 1H), 7.21 (d, *J* = 2.4 Hz, 1H), 7.38 (s, 1H), 7.40 (s, 1H), 7.43–7.45 (m, 1H), 7.49 (dd, *J* = 8.8 and 2 Hz, 1H), 7.95 (dd, *J* = 8.0 and 2.0 Hz, 1H), 8.42 (d, *J* = 8.0 Hz, 1H), 8.51 (s, 2H), 8.80 (d, *J* = 2.4 Hz, 1H), 9.21 (s, 1H). ^13^C-NMR (DMSO*-d*_6_) *δ* 55.62, 56.52, 60.63, 97.26, 114.56, 115.98, 120.25, 120.37, 122.61, 124.28, 131.05, 131.63, 133.04, 134.07, 134.44, 135.29, 146.17, 147.69, 148.53, 152.75, 152.89, 154.15, 155.02.

*1-(2-Fluorophenyl)-3-(4-(pyridin-3-yl)-2-(3,4,5-trimethoxyphenoxy)phenyl)urea* (**5h**). Yield: 0.103 g (74%). m.p.: 103–106 °C. IR (KBr) 3424, 3030, 2938, 1707, 1533, 822 cm^−1^. ^1^H-NMR (DMSO*-d*_6_) *δ* 3.67 (s, 3H), 3.76 (s, 6H), 6.50 (s, 2H), 7.04–7.08 (m, 1H), 7.25–7.28 (m, 3H), 7.43 (dd, *J* = 4.8 and 8.0 Hz, 1H), 7.51 (dd, *J* = 8.4 and 2.0 Hz, 1H), 7.96 (td, *J* = 8.0 and 2.0 Hz, 1H), 8.22 (dt, *J* = 1.6 and 8.4 Hz, 1H), 8.40 (d, *J* = 8.8 Hz, 1H), 8.52 (dd, *J* = 4.8 and 1.6 Hz, 1H), 8.80 (d, *J* = 2.4 Hz, 1H), 9.12 (s, 1H), 9.31 (d, *J* = 1.6 Hz, 1H).

*1-(2,4-Dichlorophenyl)-3-(4-(pyridin-3-yl)-2-(3,4,5-trimethoxyphenoxy)phenyl)urea* (**5i**). Yield: 0.12 g (78%). m.p.: 186–188 °C. IR (KBr) 3333, 3096, 2938, 1712, 1591, 806 cm^−1^. ^1^H-NMR (DMSO*-d*_6_) *δ* 3.67 (s, 3H), 3.75 (s, 6H), 6.50 (s, 2H), 7.20 (d, *J* = 2.0 Hz, 1H), 7.30 (dd, *J* = 2.8 and 8.8 Hz, 1H), 7.42 (dd, *J* = 4.8 and 8 Hz, 1H), 7.50 (dd, *J* = 8.8 and 2.8 Hz, 1H), 7.43 (d, *J* = 8.8 Hz, 1H), 7.93 (d, *J* = 2.8 Hz, 1H), 7.95 (d, *J* = 4.4 Hz, 1H), 8.37 (d, *J* = 8.4 Hz, 1H), 8.52 (dd, *J* = 4.4 and 1.6 Hz, 1H), 8.68 (s, 1H), 8.80 (d, *J* = 2.4 Hz, 1H).

*1-(4-Chloro-3-(trifluoromethyl)phenyl)-3-(4-(pyridin-3-yl)-2-(3,4,5-trimethoxyphenoxy)phenyl)urea* (**5j**). Yield: 0.144 g (88%). m.p.: 110–112 °C. IR (KBr) 3333, 3070, 2940, 1711, 1595, 822 cm^−1^. ^1^H-NMR (DMSO*-d*_6_) *δ* 3.67 (s, 3H), 3.76 (s, 6H), 6.51 (s, 2H), 7.21 (d, *J* = 2.4 Hz, 1H), 7.42 (dd, *J* = 4.4 and 8.0 Hz, 1H), 7.51 (dd, *J* = 2.4 and 8.4 Hz, 1H), 7.62 (s, 1H), 7.95 (dt, *J* = 2.4 and 6 Hz, 1H), 8.11 (s, 1H), 8.38 (d, *J* = 8.8, 1H), 8.51 (d, *J* = 4.4 Hz, 1H), 8.69 (s, 1H), 8.79 (d, *J* = 2.4 Hz, 1H), 9.82 (s, 1H). ^13^C-NMR (DMSO*-d*_6_) *δ* 56.54, 60.60, 97.42, 115.96, 116.93, 116.99, 120.78, 122.58, 122.93, 123.17, 124.25, 130.79, 131.92, 132.55, 134.12, 134.61, 135.19, 139.60, 146.62, 147.73, 148.63, 152.60, 154.18.

*1-(2-Fluorophenyl)-3-(2-(4-(2-hydroxyethyl)piperazin-1-yl)-4-(pyridin-3-yl)phenyl)urea* (**5k**)*.* Yield: 0.105 g (72%). m.p.: 95–97 °C. IR (KBr) 3303, 3042, 2923, 1728, 1525, 804 cm^−1^. ^1^H-NMR (DMSO-*d*_6_) *δ* 2.73–2.75 (m, 6H), 2.95 (s, 4H), 4.26 (t, *J* = 5.6 Hz, 2H), 7.15–7.17 (m, 4H), 7.43–7.45 (m, 2H), 8.10–8.13 (m, 2H), 8.52 (s, 1H), 8.54 (d, *J* = 4.8 Hz, 1H), 8.90 (s, 1H), 8.91 (s, 1H), 9.36 (s, 1H).

*1-(4-Methylphenyl)-3-(2-(4-(2-hydroxyethyl)piperazin-1-yl)-4-(pyridin-3- yl)phenyl)urea* (**5l**). Yield: 0.102 g (70%). m.p.: 166–168 °C. IR (KBr) 3306, 3473, 3031, 2923, 1707, 1517, 813 cm^−1^. ^1^H-NMR (CD_3_OD) *δ* 2.33 (s, 3H), 2.79–2.82 (m, 6H), 2.97 (t, *J* = 4.4 Hz, 4H), 4.34 (t, *J* = 5.6 Hz, 2H), 7.09 (d, *J* = 8.4 Hz, 2H), 7.16 (d, *J* = 8.4 Hz, 2H), 7.32–7.36 (m, 3H), 7.41 (d, *J* = 2.0 Hz, 1H), 7.49 (s, 1H), 8.06 (dt, *J* = 2.0 and 8.4 Hz, 1H), 8.23 (d, *J* = 8.4 Hz, 1H), 8.48 (d, *J* = 4.4 Hz, 1H), 8.79 (s, 1H).

*1-(2,4-Dichlorophenyl)-3-(2-(4-(2-hydroxyethyl)piperazin-1-yl)-4-(pyridin-3-yl)phenyl)urea* (**5m**). Yield: 0.14 g (88%). m.p.: 203–205 °C. IR (KBr) 3310, 3582, 2921, 1729, 1522, 806 cm^−1^. ^1^H-NMR (CD_3_OD) *δ* 2.86–2.91 (m, 6H), 3.01 (t, *J* = 4.4 Hz, 4H), 4.38 (t, *J* = 5.6 Hz, 2H), 7.32 (d, *J* = 2.4 Hz, 1H), 7.34 (d, *J* = 2.4 Hz, 1H), 7.42–7.46 (m, 3H), 7.51–7.54 (m, 1H), 7.76 (d, *J* = 2.4 Hz, 1H), 7.84 (d, *J* = 2.4 Hz, 1H), 8.06 (td, *J* = 1.6 and 8.0 Hz, 1H), 8.22 (d, *J* = 8.8 Hz, 1H), 8.49 (t, *J* = 3.2 Hz, 1H), 8.78 (s, 1H).

*1-(4-Chloro-3-(trifluoromethyl)phenyl)-3-(2-(4-(2-hydroxyethyl)piperazin-1-yl)-4-(pyridin-3-yl)phenyl)urea* (**5n**). Yield: 0.155 g (91%). m.p.: 120–122 °C. IR (KBr) 3305, 3441, 3052, 2931, 1728, 1524, 830 cm^−1^. ^1^H-NMR (DMSO-*d*_6_) *δ* 2.75 (t, *J* = 5.6 Hz, 2H), 2.81 (s, 4H), 2.93 (s, 4H), 4.38 (t, *J* = 5.6 Hz, 2H), 7.46–7.48 (m, 1H), 7.48–7.49 (m, 1H), 7.51 (d, *J* = 2.4 Hz, 1H), 7.62 (s, 1H), 7.64 (s, 1H), 7.74 (d, *J* = 2.4 Hz, 1H), 7.76 (d, *J* = 2.4 Hz, 1H), 8.06 (s, 1H), 8.15 (d, *J* = 2.4 Hz, 1H), 8.29 (s, 1H), 8.53 (dd, *J* = 1.6 and 4.8 Hz, 1H), 8.90 (dd, *J* = 2.4 and 0.4 Hz, 1H).

## 4. Conclusions

A new series of 1-phenyl-3-(4-(pyridin-3-yl)phenyl)urea derivatives have been synthesized and biologically evaluated as antiproliferative agents against nine different cancer cell lines. The present investigation highlights the significance of adding hydrogen bond moiety on the titled scaffold. Compounds **5a**–**e** with a hydroxymethyl piperidine moiety exhibited superior antiproliferative activity than paclitaxel and gefitinib against the most sensitive cell lines. Among the five compounds, compounds, **5a** and **5d** showed promising mean growth inhibitions with significant efficacies and superior potencies than paclitaxel and gefitinib in different cancer cell lines, particularly ones belonging to melanoma cell lines. Moreover, compound **5a** elicited lethal rather than inhibitory effects on the SK-MEL-5 melanoma, 786-0, A498, RXF 393 renal cancer, and MDA-MB-468 breast cancer cell lines. The findings of present study point towards the potential of 1-phenyl-3-(4-(pyridin-3-yl)phenyl)urea derivatives as promising leads for future development of broad spectrum anticancer agents.

## Figures and Tables

**Table 1:** Percentage growth inhibition results exerted by compounds **5a**–**e** over the most sensitive cell lines.

Cell Lines		Percentage Inhibition (at 10 μM)				
		5a	5b	5c	5d	5e
**Leukemia**	**CCRF-CEM**				90.3	82.2
	**HL-60(TB)**	86.1			**106.9**	93.7
	**K-562**	90.6			84.9	82.3
	**MOLT-4**	82.4			95.2	**100.6**
	**RPMI-8226**	80.6			87.9	90.8
	**SR**	82.9			97.7	93.4
**NSCLC**	**A549/ATCC**	93.0				
	**NCI-H23**	96.0				
	**NCI-H460**				81.5	
	**NCI-H522**	90.7				
**Colon cancer**	**COLO 205**				**126.6**	83.9
	**HCC-2998**	85.9			86.1	
	**HCT-116**	91.6		87.5	88.2	81.9
	**HCT-15**				85.2	
	**HT29**	94.3		90.2	90.8	
	**KM12**					
	**SW-620**					82.4
**CNS cancer**	**SF-295**				82.2	
	**SF-539**	98.7		84.9	81.1	
	**SNB-19**	84.7				
	**U251**	88.7				
**Melanoma**	**MDA-MB-435**	95.7			84.5	
	**SK-MEL-2**				**110.3**	
	**SK-MEL-28**	90.6		81.3		
	**SK-MEL-5**	**146.1**	80.0	**138.3**	88.7	
	**UACC-62**	95.3				
**Ovarian cancer**	**OVCAR-3**	95.3				
	**SK-OV-3**					95.7
**Renal Cancer**	**786-0**	**108.7**			84.4	
	**A498**	**136.2**		90.9	85.7	93.6
	**ACHN**				82.7	
	**RXF 393**	**134.8**				
	**TK-10**	83.5				
	**UO-31**					83.0
**Prostate Cancer**	**PC-3**				87.6	83.4
**Breast Cancer**	**MCF7**	90.2			83.8	80.0
	**MDA-MB-231/ATCC**	90.7			**117.5**	86.4
	**HS 578T**	89.4				
	**T-47D**				94.4	82.5
	**MDA-MB-468**	**116.6**			87.4	

The bold figures indicate lethal effects in terms of percentage.

**Table 2:** Mean percentage growth of the 60 cell lines after treatment with tested target compounds (10 µM).

Comp. No.	Mean % Growth
**5a**	**22**
**5b**	59
**5c**	53
**5d**	**24**
**5e**	**32**
**5f**	99
**5g**	97

The bold figures indicate the most active compound.
